# Chinese herbal medicines compared with *N*-acetylcysteine for the treatment of idiopathic pulmonary fibrosis: Protocol for a systematic review

**DOI:** 10.1097/MD.0000000000013077

**Published:** 2018-11-02

**Authors:** Jing Guo, Bin Li, Wenyuan Li, Yi Pan, Zhichao Wang, Yuxiao Wu, Fei Wang

**Affiliations:** a Chengdu University of Traditional Chinese Medicine, Chengdu, Sichuan; b Centre for Evidence-Based Chinese Medicine, Beijing University of Chinese Medicine, Beijing, China.

**Keywords:** Chinese herbal medicines, idiopathic pulmonary fibrosis, *N*-acetylcysteine, protocol, systematic review

## Abstract

**Background:**

Idiopathic pulmonary fibrosis (IPF) is a major public health problem worldwide. There is no curative treatment for IPF except lung transplantation. Chinese herbal medicines (CHMs) are widely used in the treatment of IPF in China. However, their effectiveness and safety are still obscure and deserve further investigation. The aim of the study was to assess the efficacy and safety of CHMs in treating IPF compared with *N*-acetylcysteine (NAC).

**Methods:**

This review summarizes and meta-analyzes randomized controlled trials (RCTs) of CHMs for the treatment of IPF. RCTs compare either CHMs alone or in combination with NAC or conventional medicine treatment (CMT) vs NAC alone or in combination with CMT have been included. The following electronic databases have been searched: PubMed, Cochrane Library, Embase, CNKI, CBM, VIP, and WANFANG DATA. The methodologic quality of RCTs has been assessed using the Cochrane risk assessment tool. All trials included are analyzed according to the criteria of the Cochrane Handbook. Review Manager 5.3, R-3.5.1 software, and GRADE pro GDT web solution are used for data synthesis and analysis.

**Results:**

This review evaluates the effects of CHMs on acute exacerbation, mortality, the quality of life, 6-minute walking test distance, lung function (total lung capacity, diffusing capacity of the lungs for carbon monoxide, and forced vital capacity), partial pressure of oxygen in blood (PaO_2_), and safety in patients with IPF.

**Conclusion:**

This review provides clear evidence to assess the effectiveness and safety of CHMs for IPF.

## 1 Introduction

Idiopathic pulmonary fibrosis (IPF) is defined as a special type of chronic, progressive, interstitial lung disease of unknown etiology. IPF occurs in the elderly and the lesion is confined to the lungs. Adults with chronic labor dyspnea due to unexplained causes, and the clinical manifestations of cough, lung bases *Velcro* rale and clubbing of fingers should be considered the potential patients with IPF. Progression of IPF is characterized by increased respiratory symptoms such as dyspnea and cough, worsening lung function, and High Resolution CT with increased fibrosis or acute respiratory failure. The incidence of IPF increases with age. Typical symptoms usually appear in the 60 to 70 age group. More men have been reported with IPF than women, and most patients have a history of smoking.^[1]^ One study showed that the cumulative prevalence of IPF increased from 202 to 495 cases per 100,000 people aged 65 years and older from the years 2001 to 2011.^[2]^ Due to its rapid progress, poor prognosis, and high mortality, IPF has been called a refractory lung disease. The median survival time from respiratory symptoms to death is 2 to 3 years.^[3–5]^ It has a very poor prognosis with a 5-year survival rate of 30% or below.^[6]^ The patient's survival rate is similar to that of a malignant tumor, which seriously threatens the health of human beings. Increasing rates of hospital admissions and deaths due to IPF also suggest an increasing burden of disease.^[7–10]^

The pathogenesis of IPF is still unclear, and there is no definitive conclusion at home and abroad. In addition to lung transplantation, there is no specific treatment for IPF in Western medicine, and lung transplantation is also only available for a small number of patients who can tolerate surgery. As a result, there is currently no consensus on the optimal treatment of IPF. Unfortunately, there is no evidence to prove which drug is effective in treating the disease, and only a few studies suggest that certain drugs may be beneficial for IPF.^[1]^ At present, glucocorticoids and immunosuppressive agents are widely used in clinical treatment, but their curative effect is not exact, what is worse, it is easy to form dependence with many side effects. The evidence-based guidelines of IPF have strongly rejected these therapies, so the prevention and treatment of IPF have become a problem in today's medical research. The 2015 edition of IPF's diagnostic and therapeutic guideline lists *N*-acetylcysteine (NAC) treatment as a weak and unrecommended level.^[11]^ Although the guideline suggests that IPF should not to be treated with NAC, NAC monotherapy can improve mental health and 6-minute walking distance.^[12]^ Therefore, patients who have started NAC monotherapy are not recommended to discontinue treatment.^[11,13]^ Although Pirfenidone and Nintedanib administration have been shown to delay the decline in lung function, they are rarely used clinically in China because of their significant adverse events and long-term economic costs. Therefore, NAC is widely used in the treatment of IPF because of its low price, good tolerance, and easy oral administration.^[14]^ However, due to the lack of evidence-based medical research, its safety and effectiveness remain controversial.

Today, traditional Chinese Medicine (TCM), as an adjuvant therapy to Western medicine, has its unique superiority and proper effect and is still used extensively for the treatment of IPF in China.^[15]^ Hundreds of different herbs have been used in numerous TCM formulations to treat IPF. These TCM formulations are derived from the wisdom of Chinese clinicians and the experience accumulated over thousands of years of using these herbs. In recent years, the experimental and clinical research on the treatment of IPF by TCM has gradually increased. Recent studies have shown that TCM treatment of IPF has become feasible and provide new ideas for solving the controversial problem of selecting effective drugs. It is important to highlight that more and more formulas, herbal compounds, and single herb extracts were proved to have an effect in preclinical experimental studies of IPF.^[16–18]^ TCM is a potential therapy for IPF due to its advantages of syndrome differentiation and treatment, small adverse reactions, slow and long-lasting effects, overall regulation, and no obvious drug dependence. A widespread strategy underlying the TCM treatment of IPF is to effectively relieve clinical symptoms and improve the quality of life (QOL) and reduce mortality of the patients.^[19–21]^ However, due to the small sample size, large difference of intervention measures, and the comparison of glucocorticoid not recommended in the guidelines, the evidence for the efficacy and safety evaluation of TCM treatment for IPF is insufficient.

The goals of this systematic review are to obtain evidence on the efficacy and safety of CHMs compared with NAC for IPF.

## 2 Methods

This study has been registered in PROSPERO (http://www.crd.york.ac.uk/PROSPERO), registration number: CRD 42018105348. The procedure of this protocol is based on PRISMA-P guidance.^[22]^

### 2.1 Database and search strategy

The following databases have been searched: 3 English medical databases (Cochrane Library, PubMed, and EMBASE) and 4 Chinese medical databases (China National Knowledge Infrastructure Database [CNKI], Chinese Biomedical Literature Database [CBM], VIP Chinese Science and Technology Periodical Database [VIP], and Wan Fang Data). The databases are extensively searched from their inceptions up to June 4, 2018. The search strategy is based on the guidance of the Cochrane Handbook. Terms searched include: (Traditional Chinese medicine OR Chinese medicinal OR Chinese medicine OR Chinese herbal medicine OR decoction OR Chinese patent medicine OR Chinese medicine preparation OR integration of Chinese and Western medicine) AND (Idiopathic pulmonary fibrosis OR pulmonary fibrosis OR pulmonary interstitial fibrosis OR Idiopathic pulmonary interstitial fibrosis OR IPF OR PF) AND (random∗). To guarantee comprehensive search, all relevant publications are researched, including academic dissertation and conference articles. The language is limited to Chinese and English.

### 2.2 Inclusion criteria

#### 2.2.1 Types of studies

Only randomized controlled trials (RCTs) are included.

#### 2.2.2 Types of participants

Male or female patients of any age or ethnic origin who have been diagnosed with IPF have been included. Diagnostic criteria refer to the 2011 international evidence-based guidelines for IPF diagnosis and treatment jointly published by ATS/ERS/JRS/ALAT.^[1]^ In addition, we also refer to the consensus of Chinese experts on IPF diagnosis and treatment formulate by the interstitial pulmonary disease group of the Chinese academy of respiratory medicine in 2016.^[23]^

#### 2.2.3 Types of intervention

The experimental group is treated with Chinese herbal medicines (CHMs; or combine with conventional medicine treatment), the control group is treated with NAC, and both groups are treated orally with a treatment period of not <12 weeks. CHMs include Chinese medicine decoction, Chinese medicine preparation (such as tablet, pill, powder, cream, Dan, and so on), Chinese medicine single prescription, Chinese medicine compound prescription, and Chinese patent medicine. Both groups can receive the same routine western medicine treatment. Basic medical treatment of Western medicine includes oxygen therapy, infection control, and nutritional support.

#### 2.2.4 Types of outcome measures

Primary outcomes: The rate of acute exacerbation and mortality after treatment and the health-related QOL have been analyzed as the primary outcome. The health-related QOL has been assessed using St George's respiratory questionnaire scores.

Secondary outcomes: The secondary outcome assessments consist of pulmonary function test—especially forced vital capacity (FVC), total lung capacity (TLC), the diffusing capacity of the lungs for carbon monoxide (DLCO), 6-minute walk test (6MWT) distance, partial pressure of oxygen in blood (PaO_2_), and adverse events. The time endpoint of the above outcomes is no earlier than 12 weeks after starting the medication.

### 2.3 Exclusion criteria

1. The unrelated and duplicated documents have been deleted.
2. Animal experiments, reviews, theoretical discussions, experience summaries, and case reports.
3. The control drug in the studies is not NAC.
4. The drugs used in included studies are not oral administration.
5. Review articles without original data.

### 2.4 Data collection and extraction

Referring to the Cochrane collaborative network system evaluator handbook^[24]^: importing the search results into the document management software of NoteExpress (version:3.2; Beijing Aegean Software Company, Beijing, China); excluding the duplicate literature using NoteExpress3.2 and excluding the unrelated articles by reading the title and abstract; and reading the full text and reserving clinical studies that meet the inclusion criteria. Two researchers (GJ and LWY) extract the data independently using a self-developed data extraction form. The differences encountered in the process have been resolved by discussing with another team member (LB), to determine, by agreement, the final selection of studies.

Data extraction contents include: general information: research ID, author, title, publication status, report sources, and fund support; methodology information: design, number of arms, random sequence generation, allocation concealment, blinding, incomplete outcome data, selective reporting, sample size calculation, and baseline comparability; participant information: diagnostic criteria, inclusion criteria, exclusion criteria, setting, population, sample size, age, gender, and course of disease; intervention information: name of intervention and comparation, syndrome differentiation of TCM, types of CHMs, dosage form, comparison, duration of treatment, and patient follow-up; outcomes; and adverse events.

**Figure 1:**
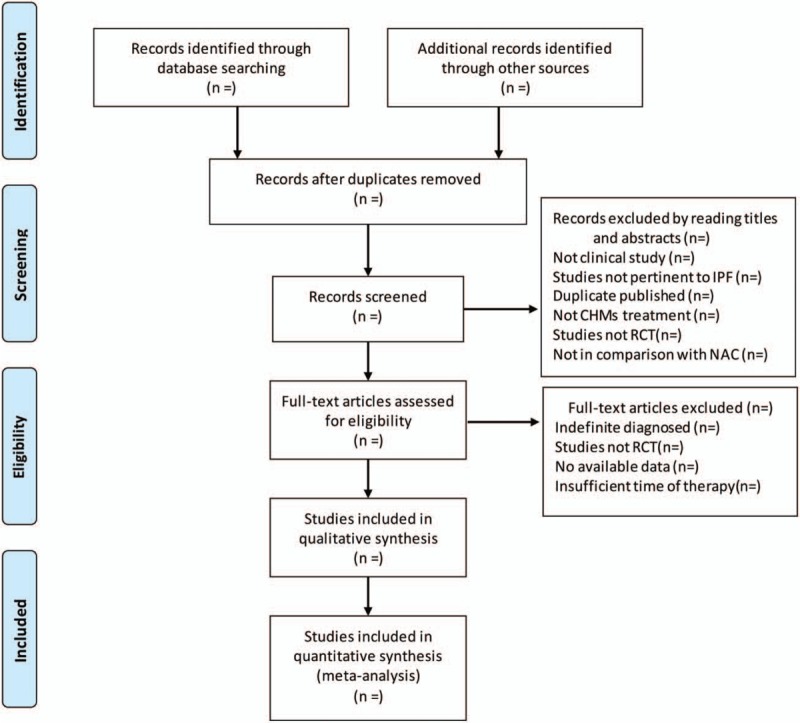
Flow chart of the selection process.^[31]^ Arrows = flow directions or reasons of trials exclusion; CHMs = Chinese herbal medicines; IPF = idiopathic pulmonary fibrosis; NAC = *N*-acetylcysteine; RCT = randomized controlled trial.

The selection process was showed in a PRISMA flow chart (http://www.prisma-statement.org/) (Fig. 1).

### 2.5 Assessment of methodologic quality

The Cochrane risk assessment tool has been used.^[25]^ Risk of bias has been assessed as follows: adequacy of generation of the allocation sequence, allocation concealment, double blinding, incomplete outcome date, selective outcome reporting, follow-up, and other bias. These domains classify “Yes” if adequate, “No” if not adequate, and “Unclear” if not well described by the authors in such a way that its adequacy is describable.

The 2 researchers (GJ and LWY) independently assessed the risk of bias for each included study. “L,” “H,” and “U” have been used as a code for the evaluations of the above bias risks. “L” indicates a low risk of bias, “H” indicates a high risk of bias, and “U” indicates the risk of bias is unclear. Disagreements resolved by discussion between all the researchers. When necessary, the study authors have been contacted to inquire some missing information. Trials of high risk of bias will be considered when conducting sensitive analysis.

### 2.6 Data synthesis and analysis

Review Manager Software (RevMan, Version 5.3 for windows, The Cochrane Collaboration, Oxford, England) has been used to analyze and synthesize the outcomes. Clinical heterogeneity is the primary source of heterogeneity in the systematic reviews of TCM. Clinical heterogeneity can be derived from the potential factors such as the ingredients and formulations of CHMs.^[26]^ Quantitative synthesis has been done when clinical heterogeneity is not considered by at least 2 authors in discussion. Continuous variable has been described by mean difference (MD), *P*–value, and 95% confidence interval (CI). For dichotomous outcomes, the relative risk (RR) has been used, with 95% CI and *P*-values, to evaluate the efficacy and safety of CHMs. *I*^2^ test has been used to judge the heterogeneity of meta-analysis. *I*^2^ value >50% or more will be considered as an indication of substantial heterogeneity. If heterogeneity exists in the pooled studies, the data have been analyzed using a random effects model. Otherwise, a fixed effect model has been adopted. If there is significant clinical heterogeneity, the cause of heterogeneity should be explored, and sensitivity analysis or subgroup analysis should be performed when necessary. Sensitivity analysis has been used to ensure the robustness of results by eliminating low-quality trials. Subgroup analysis will be performed according to the characteristics of the study subjects, such as different interventions, treatment duration, and outcome measures. If the data extraction is insufficient, qualitative analysis will be adopted.

### 2.7 Publication bias

The publication bias has been analyzed using funnel plot when the number of studies included in a meta-analysis is no <10. If the number of included studies is <10, the Egger test will be applied. The analysis software is R 3.5.1 for Windows.

### 2.8 Quality of evidence

This study evaluates the evidence according to GRADE standard, which refers grading of recommendations assessment, development and evaluation. These factors that may reduce the quality of evidence should be considered, such as limitations in study design, inconsistency of results, indirectness of evidence, inaccuracies, and publication bias. In addition, large magnitude effect, possible confounders that can reduce the effect and dose-response gradient that increase the quality of evidence cannot be ignored. GRADE Pro GDT online software will be used to form the summary of findings table (SoF table).

## 3 Discussion

The CHMs have been used for thousands of years in China, and now it is widely used in the world. Currently, several types of CHM extracts are reported to be effective in animal models of IPF. The purpose of this study is to summarize and analyze the efficacy and safety of clinical trials using CHMs to treat IPF. Although there are many trials on the treatment of diseases by CHMs in China, the overall quality is not high. Comparing with the systematic review of modern medicine, there are few studies on high-quality systematic review of CHMs and lack of reasonable evaluation system leads to insufficient reliable evidence for clinical use of CHMs.^[27]^ These problems are caused by the complexity of the TCM treatment and the difficulty to clarify the heterogeneity of the scheme, resulting in the low reliability of evaluation. It is well known that CHMs in China are based on thousands of years of clinical observation and practice by Chinese doctors. In general, many herbs are used in combination to treat diseases. Each herb in Chinese medicine preparations may have several different active ingredients. The content and bioactivity of these ingredients can be affected according to the cultivation region, soil, climate and sunshine, as well as the season of cultivation and harvesting and the method of preparation. Therefore, clinical trials must provide detailed information about the CHMs used. Different from modern clinical research, it is difficult to compare the results of TCM research. Properly speaking, in the same Chinese medicine prescriptions, batch, medicinal processing, dosage, dosage form of the herbs, route of administration, and duration of therapy are all factors that lead to different characteristics of the CHMs, and more importantly, these factors will affect the treatment effect of the disease. Therefore, we believe that the combination of CHMs and modern medical technologies and therapies is the premise and foundation for understanding the potential effectiveness of CHMs in IPF treatment.

The mechanism of TCM treatment of IPF is not completely clear. One possible explanation is that the mechanism of TCM prevention and treatment of IPF mainly regulates cytokines and signal transduction pathways, inhibits extracellular matrix synthesis, and regulates oxidative stress and coagulation-fibrinolysis systems. This hypothesis has been supported by a number of recent clinical trials, which have demonstrated that CHMs for activating blood circulation and removing blood stasis, tonifying qi and nourishing Yin, clearing heat and reducing phlegm and tongluo are effective in improving pulmonary fibrosis.^[28]^ However, the pharmacologic mechanism of TCM treatment of IPF and more large-scale studies and randomized, placebo-controlled studies are needed. Furthermore, the morbidity, survival time and mortality of patients with IPF require further attention and research. It is important to highlight that this review wants to determine whether CHMs, comparing with NAC, can improve acute exacerbation, mortality, the QOL, 6MWT, lung function (TLC, DLCO, and FVC), PaO_2_ and adverse events in patients with IPF. Previous clinical studies have paid little attention to the acute exacerbation and mortality of patients with IPF. Only 1 relevant systematic review mentions the impact of CHMs on acute exacerbation and mortality in patients with IPF and concludes that CHMs can reduce the acute exacerbation rate but have no effect on mortality.^[29]^ However, due to the problems of small sample size, poor methodologic quality, and large variability of CHMs, the evidence for the result is weak. To avoid these limitations as much as possible, the clinical trials of TCM included this review will be conducted strictly and carefully and recommended to follow the CONSORT—CHM Formulas 2017.^[30]^ Besides, we also achieve important implications from this review for future high-quality clinical trials to get explicit evidence. We hope this review will stimulate proper evaluation of CHMs.

### 3.1 Ethics and dissemination

This review does not require ethical approval because the included studies are published data and do not involve the patients’ privacy. The results of this review will be reported in accordance with the PRISMA extension statement and disseminated to a peer-reviewed journal.

## Author contributions

This study is initiated by Fei Wang and Jing Guo. Fei Wang and Jing Guo were involved in the design of the study and the interventions of the protocol. Jing Guo, Bin Li, and Wenyuan Li will develop the search strategies, conduct data collection, and analyze independently. Yi Pan, Zhichao Wang, and Yuxiao Wu will revise it.

All authors have approved the final manuscript.

**Conceptualization:** Fei Wang.

**Methodology:** Jing Guo, Bin Li, Wenyuan Li, Yuxiao Wu.

**Software:** Zhichao Wang.

**Supervision:** Fei Wang.

**Writing – original draft:** Jing Guo, Bin Li.

**Writing – review & editing:** Jing Guo, Bin Li, Wenyuan Li, Yi Pan.

## References

[1] Raghu G Collard HR Egan JJ An official ATS/ERS/JRS/ALAT statement: idiopathic pulmonary fibrosis: evidence-based guidelines for diagnosis and management. Am J Respir Crit Care Med 2011;183:788–824. 21471066 10.1164/rccm.2009-040GL PMC5450933

[2] Raghu G Chen SY Yeh WS Idiopathic pulmonary fibrosis in US Medicare beneficiaries aged 65 years and older: incidence, prevalence, and survival, 2001-11. Lancet Respir Med 2014;2:566–72. 24875841 10.1016/S2213-2600(14)70101-8

[3] Collard HR King TE Jr Bartelson BB Changes in clinical and physiologic variables predict survival in idiopathic pulmonary fibrosis. Am J Respir Crit Care Med 2003;168:538–42. 12773325 10.1164/rccm.200211-1311OC

[4] Fernandez Perez ER Daniels CE Schroeder DR Incidence, prevalence, and clinical course of idiopathic pulmonary fibrosis: a population-based study. Chest 2010;137:129–37. 19749005 10.1378/chest.09-1002 PMC2803118

[5] Ley B Collard HR King TE Jr Clinical course and prediction of survival in idiopathic pulmonary fibrosis. Am J Respir Crit Care Med 2011;183:431–40. 20935110 10.1164/rccm.201006-0894CI

[6] Homma S Bando M Azuma A Japanese guideline for the treatment of idiopathic pulmonary fibrosis. Respir Investig 2018;56:268–91. 10.1016/j.resinv.2018.03.003 29980444

[7] American Thoracic Society Idiopathic pulmonary fibrosis: diagnosis and treatment. International consensus statement. American Thoracic Society (ATS), and the European Respiratory Society (ERS). Am J Respir Crit Care Med 2000;161:646–64. 10673212 10.1164/ajrccm.161.2.ats3-00

[8] Hutchinson J Fogarty A Hubbard R Global incidence and mortality of idiopathic pulmonary fibrosis: a systematic review. Eur Respir J 2015;46:795–806. 25976683 10.1183/09031936.00185114

[9] Hutchinson JP McKeever TM Fogarty AW Increasing global mortality from idiopathic pulmonary fibrosis in the twenty-first century. Ann Am Thorac Soc 2014;11:1176–85. 25165873 10.1513/AnnalsATS.201404-145OC

[10] Navaratnam V Fogarty AW Glendening R The increasing secondary care burden of idiopathic pulmonary fibrosis: hospital admission trends in England from 1998 to 2010. Chest 2013;143:1078–84. 23188187 10.1378/chest.12-0803

[11] Raghu G Rochwerg B Zhang Y An official ATS/ERS/JRS/ALAT clinical practice guideline: Treatment of idiopathic pulmonary fibrosis. An update of the 2011 clinical practice guideline. Am J Respir Crit Care Med 2015;192:e3–19. 26177183 10.1164/rccm.201506-1063ST

[12] Sun T Liu J Zhao DW Efficacy of *N*-acetylcysteine in idiopathic pulmonary fibrosis: a systematic review and meta-analysis. Medicine (Baltimore) 2016;95:1–7. 10.1097/MD.0000000000003629 PMC4902516 27175674

[13] Wijsenbeek MS Collard HR Acetylcysteine in IPF: the knockout blow? Lancet Respir Med 2016;4:420–1. 27161256 10.1016/S2213-2600(16)30085-6

[14] Raghu G Noth I Martinez F N-acetylcysteine for idiopathic pulmonary fibrosis: the door is still open. Lancet Respir Med 2017;5:e1–2. 28000596 10.1016/S2213-2600(16)30327-7

[15] Zhang Y Mao X Su J A network pharmacology-based strategy deciphers the underlying molecular mechanisms of Qixuehe capsule in the treatment of menstrual disorders. Chin Med 2017;12:23. 28835770 10.1186/s13020-017-0145-x PMC5563918

[16] Huang H Peng X Idiopathic pulmonary fibrosis: the current status of its epidemiology, diagnosis, and treatment in China. Intractable Rare Dis Res 2013;2:88–93. 25343109 10.5582/irdr.2013.v2.3.88 PMC4204549

[17] Yang J Cui Y Kolb M How useful is traditional herbal medicine for pulmonary fibrosis? Respirology 2009;14:1082–91. 19909458 10.1111/j.1440-1843.2009.01644.x

[18] Chen YY Fan XM Advances in the treatment of IPF with traditional Chinese medicine. Chin J Clin (Electronic Edition) 2010;4:130–2.

[19] Yu H Liu JP Ge JZ A systematic review on randomized controlled trial of curative effects and safety of Chinese medical preparations on pulmonary interstitial fibrosis. J Beijing Univ Tradit Chin Med 2008;15:23–8.

[20] Li GH Hao JJ Li N Clinical efficacy of traditional Chinese medicine in treatment of idiopathic pulmonary fibrosis: a meta-analysis. Chin Arch Tradit Chin Med 2018;36:898–902.

[21] Zang NZ Pang LJ Li P Establishment and evaluation of IPF-TCM-HRQOL32: a health-related quality of life of TCM scale for patients with idiopathic pulmonary fibrosis. Chin J Tradit Chin Med Pharm 2016;5:1638–42.

[22] Moher D Shamseer L Clarke M Preferred Reporting Items for Systematic Review and Meta-Analysis Protocols (PRISMA-P) 2015 statement. Syst Rev 2015;4:1. 25554246 10.1186/2046-4053-4-1 PMC4320440

[23] Interstitial Lung Diseases Group of the Chinese Medical Association Respiratory Diseases Society Chinese expert consensus on diagnosis and treatment of idiopathic pulmonary fibrosis. Chin J Tuberc Respir Dis 2016;39:421–32.

[24] Higgins JP Altman DG Gϕtzsche PC The Cochrane collaboration's tool for assessing risk of bias in random trials. BMJ 2011;343:d5928. 22008217 10.1136/bmj.d5928 PMC3196245

[25] Higgins JP Green S Cochrane Handbook for Systematic Reviews of Interventions Version 5.0.2. The Cochrane Collaboration, 2009 Available from http://handbook.cochrane.org.

[26] Hu D Kang DY Hong Q Heterogeneity analysis of systematic reviews on traditional Chinese medicine. Chin J Evid-Based Med 2010;10:488–91.

[27] Zhang JH Shang HC Gao XM Methodology and reporting quality of systematic review/meta-analysis of traditional Chinese medicine. J Altern Complement Med 2007;13:797–805. 17983335 10.1089/acm.2007.7195

[28] Yang L Lv XD Liu YM Research progress on the mechanism of oxidative stress in traditional Chinese medicine on prevention and treatment of pulmonary fibrosis. World J Integr Tradit West Med 2017;12:297–301.

[29] Wu Q Zhou Y Feng FC Effectiveness and safety of Chinese medicine for idiopathic pulmonary fibrosis: a systematic review and meta-analysis. Chin J Integr Med 2018;1–7. 10.1007/s11655-017-2429-5 29335860

[30] Cheng CW Wu TX Shang HC CONSORT Extension for Chinese Herbal Medicine Formulas 2017: recommendations, explanation, and elaboration (traditional Chinese version). Ann Intern Med 2017;167:W7–20. 28654988 10.7326/IsTranslatedFrom_M17-2977_1

[31] Moher D Liberati A Tetzlaff J The PRISMA Group Preferred reporting items for systematic reviews and meta-Analyses: the PRISMA statement. J Clin Epidemiol 2009;62:1006–21. 19631508 10.1016/j.jclinepi.2009.06.005

